# The mitochondrial genome of clearhead icefish *Protosalanx chinensis* (Salmoniformes: Salangidae)

**DOI:** 10.1080/23802359.2018.1491335

**Published:** 2018-07-11

**Authors:** Ying Liu, Chao Song, Jiazhang Chen

**Affiliations:** a Wuxi Agriculture Committee, Wuxi, China;; b Freshwater Fisheries Research Center, Chinese Academy of Fishery Sciences, Wuxi, China

**Keywords:** Mitochondrial genome, *Protosalanx chinensis*, phylogenetic analysis

## Abstract

The complete mitochondrial genome of *Protosalanx chinensis* was determined to be 16,879 bp in length. It consists of 13 protein-coding genes, 22 tRNAs, 2 ribosomal RNAs, and a control region. The gene composition and the structural arrangement of the *P*. *chinensis* complete mtDNA were identical to most of the other vertebrates. The molecular data here we presented could play a useful role to study the evolutionary relationships and population genetics of Salangidae fish.

The clearhead icefish *Protosalanx chinensis* is a small, pelagic, carnivorous species, which mainly distributes in the coastal area of the East China Sea, Yellow Sea, and Bohai Sea and their adjacent rivers and lakes in eastern Asia (Saruwatari et al. 2002). Because of its high commercial value, *P. chinensis* has been traditionally exploited in China and are also one of the most important exported fish to European, Asian and American markets (Armani et al. 2011). To facilitate the future researches of taxonomic resolution, population genetic structure and phylogeography, the complete mitochondrial genome of *P. chinensis* was determined (GenBank number MH330683).

The samples were collected from the Taihu Lake, Wuxi city, China (31°24′44″ N, 120°19′43″ E). Muscle samples were fixed in 95% ethanol and preserved in Freshwater Fisheries Research Center, Chinese Academy of Fishery Sciences. Twenty-eight pairs of PCR primers were designed to based on sequences from related fish *Neosalanx tangkahkeii* and *N*. *anderssoni*. The complete mitochondrial genome of *P. chinensis* is 16,879 bp in length. The overall nucleotide composition was A (25.5%), T (25.1%), C (31.4%), and G (18%), without a significant AT bias of 50.6%. Mitogenome database were annotated by MitoFish (Iwasaki et al. 2013). It consists of 13 typical vertebrate protein-coding genes, 22 tRNA, 2 rRNA genes and 1 control region. ND6 gene and 8 tRNA genes were encoded on the light (L) strain; the remaining genes were encoded on the heavy (H) strain. For protein-coding genes, 12 were initiated with the orthodox ATG except for COI with GTG, and they had 4 types of stop codons (TAA, TAG, TA–, and T–). All tRNAs were recognized by tRNAscan-SE1.21 (Lowe and Eddy 1997), and their length ranged from 65 to 75 bp. The 2 rRNA genes contained 943 and 1710 bp, respectively. The control region of the *P. chinensis* mitogenome was 1221 bp in length, which was slightly longer than that of *N*. *tangkahkeii* and *N*. *anderssoni,* as it showed three tandem repeat units.

Phylogenetic analysis was performed by MEGA 6.06 (Tamura et al. 2013) based on complete mitogenome sequence of *P. chinensis* and those of 10 closely related species using maximum parsimony (MP) and neighbour-joining (NJ) method with 1000 bootstrap replicates (Figure 1). Identical tree topologies were recovered from the MP and NJ analyses, and the only differences were bootstrap values. As indicated by the tree, different species from the same family clustered together (e.g. Salangidae and Osmeridae), and species from Osmeroidei formed a monophyletic group while Salmonidae positioned as an outgroup.

**Figure 1.**
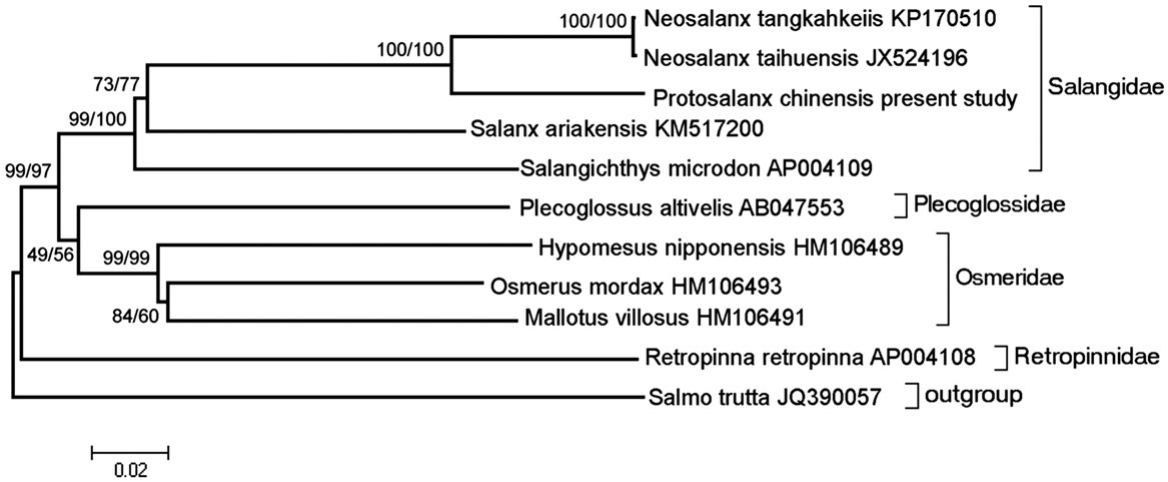
The phylogenetic relationship of *P*. *tangkahkeiis* with the other species using MP and NJ method with 1000 bootstrap replicates. The MP/NJ bootstrap support is indicated above the branch.
